# Improving Patient Satisfaction With Inhalation Sedation: A Service Evaluation of Paediatric Patients at a Primary Care Referral Centre in the United Kingdom

**DOI:** 10.7759/cureus.76025

**Published:** 2024-12-19

**Authors:** Melody Shirazi, Taneesa Javed

**Affiliations:** 1 Orthodontics, Guy's and St Thomas' NHS Foundation Trust, London, GBR; 2 Paediatrics, Birmingham Community Healthcare NHS Foundation Trust, Birmingham, GBR

**Keywords:** nitrous oxide inhalation sedation, paediatric dentistry, patients satisfaction, questionnaire, service evaluation

## Abstract

Introduction

This report explores patient satisfaction in a dental sedation service in primary care for paediatric patients. The study explores different behavioural management techniques and additional supportive aids as adjuncts to inhalation sedation to improve patient satisfaction.

Aim and objective

To determine patient satisfaction with pre-assessment, treatment and aftercare in inhalation sedation services in primary care. To improve patient satisfaction through the inclusion of behavioural management and additional supportive aids in routine practice.

Materials and methods

A retrospective service evaluation comprising 100 anonymous patient satisfaction questionnaires following treatment under inhalation sedation. The questionnaire explored satisfaction with pre-assessment, treatment and aftercare services. Routine use of various behavioural management techniques and supportive aids was introduced to improve patient and parent satisfaction.

Results

Results from two cycles showed a significant improvement in patient satisfaction following the implementation of supportive adjuncts for paediatric patients as part of the pre-assessment, treatment and post-operative care.

Conclusion

The use of pre-visit videos and other supportive aids, including scented pens, tooth pots and teddy bears wearing inhalation sedation masks, proved beneficial and significantly improved patient satisfaction.

## Introduction

Dental anxiety in paediatric patients is common, with some studies showing its prevalence to be as high as 23.9% [1]. It is typically more common in school children and pre-school children and can often lead to poorer oral health, resulting in a higher likelihood of pain and infection, thereby negatively impacting the quality of life not only for the patient but also for their family [2]. Management and treatment of this cohort of patients can often be challenging. Where behavioural management techniques and local analgesia alone cannot be used to deliver high-quality dental care to these patients, conscious sedation offers a safe and effective pharmacological alternative [3].

The most common method of conscious sedation for children in the United Kingdom (UK) is inhalation sedation using nitrous oxide and oxygen with local analgesia [3]. A titrated dose of nitrous oxide is delivered via a nasal mask, and the level of sedation can be altered based on the child's co-operation and anxiety level. This method is not only suitable for patients with significant dental anxiety and fear, but also for patients with a strong gag reflex and those with muscular tone disorders. The child must be able to cope with nasal breathing instructions for the sedation to be effective [4]. The use of behavioural management techniques should always be employed to support the delivery of inhalation sedation when treating children and young people. These techniques may include tell-show-do, enhancing control and positive reinforcement [5]. Conscious sedation can be carried out across different settings, and clinicians must have adequate training to ensure this can be provided safely. In the UK, dental services are split into the following three categories [6]: (1) Primary Care Services - these services are carried out by general dental practitioners (GDPs) based in dental practices across the UK where routine dental care is carried out. GDPs make up approximately 85% of the dental workforce in the UK [7]. (2) Secondary Care Services - most of these services are provided by hospitals within the National Health Service (NHS) and are for patients who have more complex treatment needs, including services such as complex oral surgery and paediatric care. There are several specialist dental hospitals within the UK where these services can also be delivered. (3) Community Dental Services (CDS) - these services are for adults and children who have more complex needs, such as learning or physical disabilities, individuals who require sedation and general anaesthesia to provide dental care and patients who are unable to leave their homes.

Primary dental care services also have the scope to provide more complex treatment in the form of conscious sedation on a referral basis. The Intercollegiate Advisory Committee for Sedation in Dentistry (IACSD) guidelines [8] suggest that ‘primary care dental practitioners can provide inhalation sedation for all ages in primary care.’ A referral to CDS/secondary care is more suitable for patients who require additional support and care from teams with specialists and consultants.

This report presents a service evaluation, which assesses the satisfaction of parents and patients during their inhalation sedation treatment journey. The study was carried out in a well-established NHS primary care referral centre in the UK for treatments such as restorative care, extractions under sedation and an intermediate minor oral surgery service. In this practice, patients can be referred for treatment under intravenous sedation or inhalation sedation, with most of the latter being paediatric referrals.

Once the referrals have been sent from the GDP, the referrals are triaged and the patients are pre-assessed via a telephone consultation by an appropriately trained clinician. During this pre-assessment, a history is taken and the treatment plan provided by the GDP is discussed. Different treatment modalities are discussed including treatment under local anaesthetic, inhalation sedation and intravenous sedation (for patients over the age of 12) with their associated risks and benefits. Referral to CDS and secondary care is also discussed with parents and patients as an alternative. This is mainly if they want to explore the option of general anaesthesia or if their child has special needs or a complex medical history. If the parent opts for inhalation sedation, it is further discussed that a degree of compliance is required as the patient would not be put completely to sleep and would be required to follow simple instructions when receiving treatment [3]. The potential co-operation of the patient is based on their parent's opinion, comments from the GDP on the referral as well as the patient’s behaviour in past dental visits such as their ability to sit in the chair and allow for an examination.

Following pre-assessment, the patient is contacted by the reception booking team to arrange an appointment for treatment. An email confirmation is sent including a summary of information on what to expect on the day and a link to a video made by our team, which consists of a roleplay of inhalation sedation [9]. On the day of treatment, the procedure is discussed with the parent and patient and verbal and written consent is obtained. All surgeries are fitted with televisions set to an age-appropriate channel. Following treatment, the child and parent are taken into the recovery room where a recovery nurse discusses aftercare with them. Once the treatment session is completed, there are three possible outcomes: (1) if the treatment plan was not completed, they are booked in for further appointments where the remainder of the items are completed; (2) if the treatment plan was completed, they are discharged back to their GDP; (3) if there was limited co-operation and treatment could not be completed, the patient is discharged by us back to their GDP and referred to CDS or secondary care as deemed appropriate [6].

Within the practice, there is a well-rounded, adequately trained team consisting of two sedation-trained dentists, seven sedation-trained dental nurses and one medically qualified anaesthetist. The sedation nurses assist both during the procedure and in recovery, and the anaesthetist supports medically and socially complex patients. There were no changes in the team between the two cycles.

## Materials and methods

**Figure 1:**
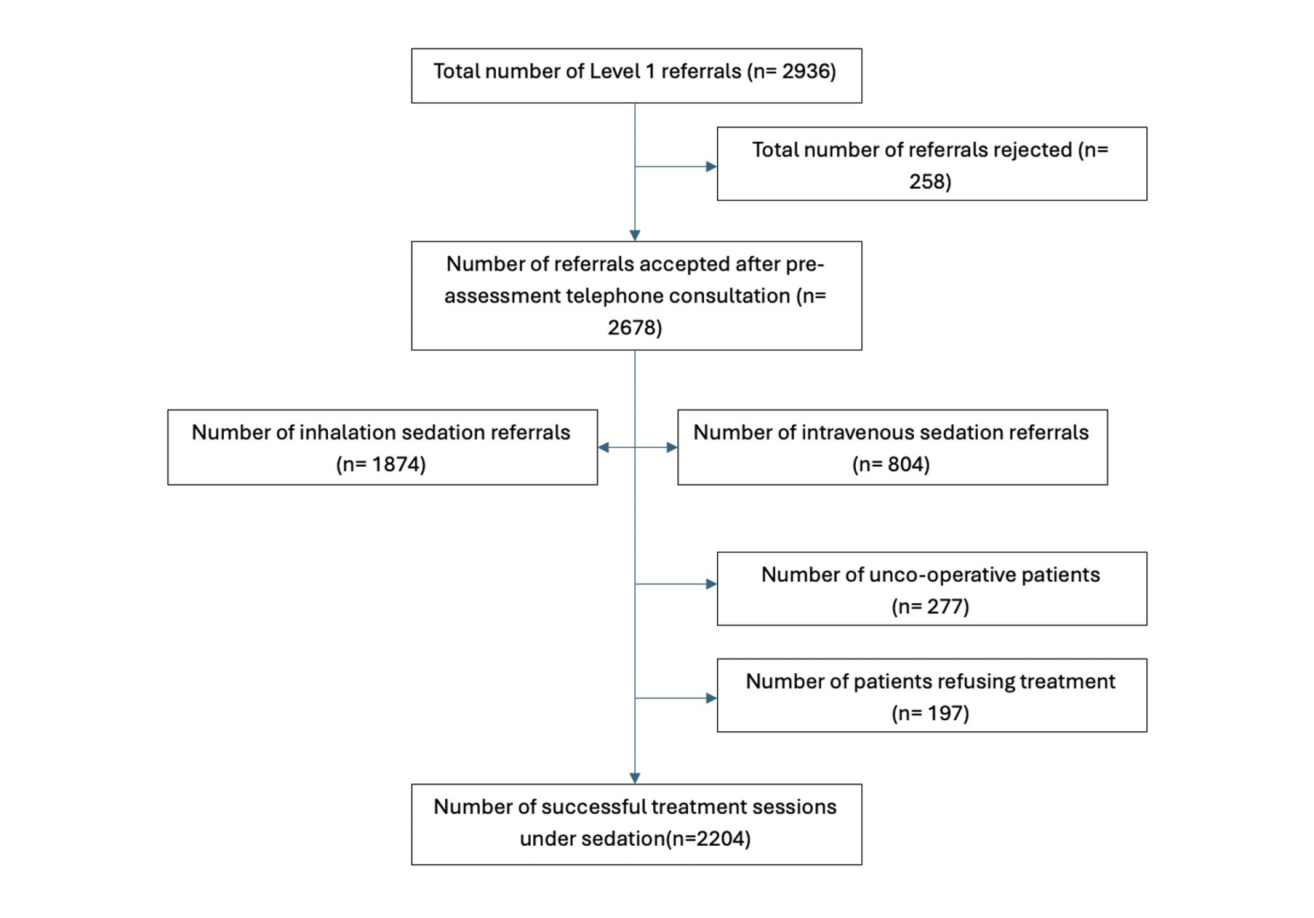
Flowchart showing the number of referrals from April 2023 to April 2024.

Between April 2023 and April 2024, a large primary care sedation referral centre in Bedfordshire, UK, received nearly 3000 sedation referrals. Figure 1 depicts a flowchart of how these referrals are triaged, and the overall number of successful treatments carried out. Of the 2936 referrals, 258 were rejected at the pre-assessment stage due to two main reasons: (1) The clinician and/or parent anticipated the child would not co-operate for treatment under conscious sedation, due to their medical, social and dental history. (2) The parents preferred general anaesthesia and opted to explore this further in a secondary care setting.

Following pre-assessment, 2678 patients were accepted for treatment, with the majority of these cases being inhalation sedation. Further to this, 474 patients were either unable to complete treatment due to poor co-operation or refused treatment on the day and, hence, were discharged back to their GDP. In approximately 75% of the referrals received by the practice, treatment under conscious sedation was carried out successfully.

Due to the high number of inhalation sedation cases seen in this practice, it was key to find out how satisfied our patients are with the service provided. Our study aimed to determine patient satisfaction with pre-assessment, treatment and aftercare to improve patient experience with the inclusion of behaviour management and additional supportive aids into routine practice.

Children up to the age of 16 receiving inhalation sedation were included in the study. Patients above the age of 16 were excluded. Any total refusal of treatment was also excluded; this included any child who refused to enter the surgery or sit in the dental chair. Patients who were graded a III and above as per the American Society of Anaesthesiologists (ASA) scoring system were excluded, as they were on separate lists with an anaesthetist present. Socially challenging patients, such as those with a language barrier, were also excluded.

The approval of the institution's clinical governance team was gained for this service evaluation, and ethical approval was not required. Questionnaires were completed anonymously; participants were provided with detailed verbal information about the study, and completion implied consent. Those who chose to participate understood that the data would be anonymously used for the results in this study.

Paper questionnaires were handed out consecutively over a five-day period to 100 parents attending with their child, following treatment under inhalation sedation, post-recovery, as highlighted in Appendix 1. The first cycle was carried out from January 15, 2024, to January 19, 2024, and the second cycle was carried out from August 12, 2024, to August 16, 2024. The questions were designed to assess both parent and patient satisfaction through the inhalation sedation journey, from pre-assessment to recovery and aftercare. It facilitated both quantitative and qualitative analyses, including a Likert scale format of questions. A free-text section was also added, encouraging participants to comment on what went well and any suggestions for improvement. At the time of this study, there is no valid or reliable questionnaire for measuring patient satisfaction with paediatric inhalation sedation for dental procedures. This questionnaire was designed with input from paediatric specialists in the field, patients and parents. The questionnaire was designed based on the entire pre-, peri- and post-operative experience, and the questions were based on existing questionnaires in the specialty of conscious sedation [10]. Simple, easy-to-understand language was used, and a five-point Likert scale was used for several questions to gain unbiased answers. Questions in this study may be taken and included in a validated assessment tool.

**Figure 2:**
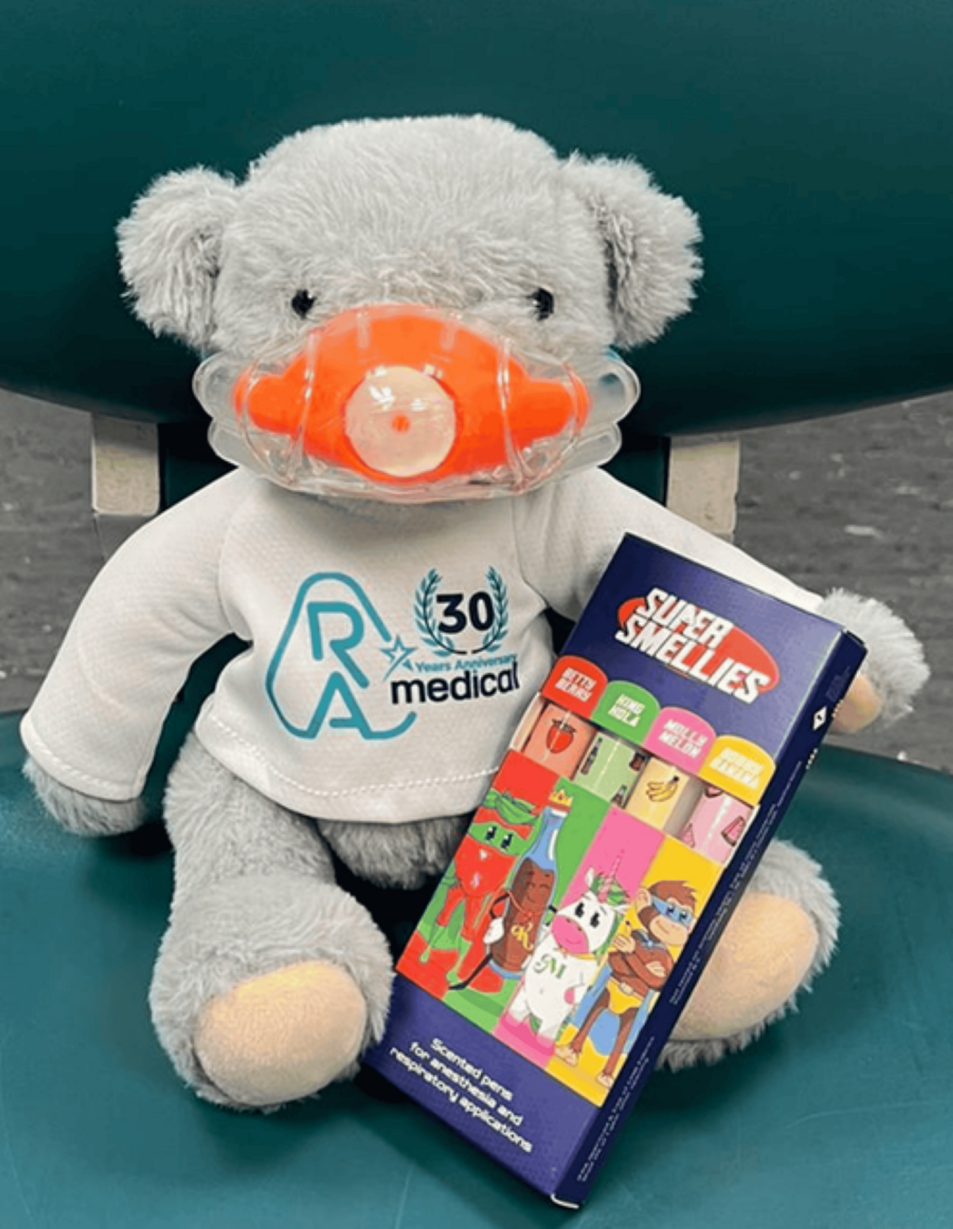
A teddy bear with an inhalation sedation mask and inhalation sedation mask scented pens.

**Figure 3:**
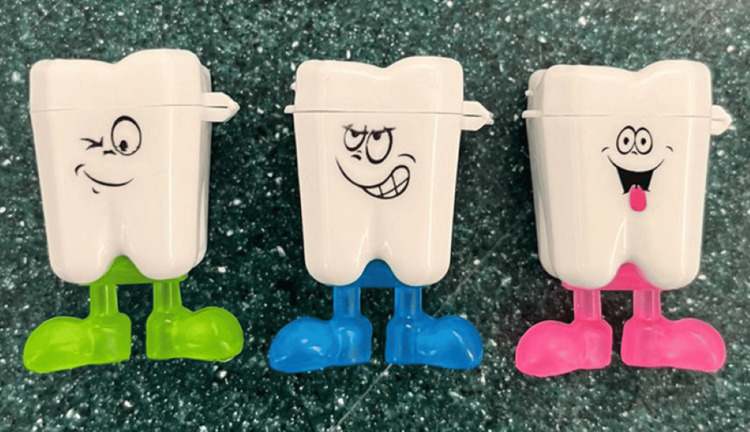
Tooth pots given to patients’ undergoing extraction to keep their teeth.

Following cycle 1, the results were analysed, and the following action plan was devised: (1) During the pre-assessment, patients were routinely advised to watch the pre-visit video [9]. (2) A teddy bear with an inhalation sedation mask was shown to patients pre-treatment (Figure 2). (3) Inhalation sedation mask-scented pens were used, as chosen by the patient, before placement of the mask (Figure 2). (4) Tooth pots were given to patients undergoing extraction to keep their teeth (Figure 3).

After two months of the above interventions, a second round of questionnaires was distributed, and the same principles of completing the questionnaires were followed. Questionnaires were all completed on paper, and results were uploaded onto an electronic spreadsheet where they were analysed. All questionnaires were completed in their entirety. The data were analysed with descriptive statistical analysis, summarising and presenting the data in bar chart form. Results from the second cycle were compared against those from the first cycle.

## Results

In both cycles, 100 patients were surveyed. The age range in cycle 1 was between 4 and 15 years of age, and in cycle 2, it was between 4 and 14 years of age. In cycle 1, 48 patients attended for extractions, 32 for fillings and 20 for fillings and extractions. In cycle 2, 40 attended for extractions, 31 for fillings and 29 for fillings and extractions. In cycle 1, 47 had watched the pre-visit video, compared to 79 in cycle 2, highlighting an increase of 32%.

Following the implemented actions, there was greater satisfaction in the experience of parents and patients. As a result, 100% of questions showed improvement from the first to the second cycle regarding the inhalation sedation service provided by the practice.

**Figure 4:**
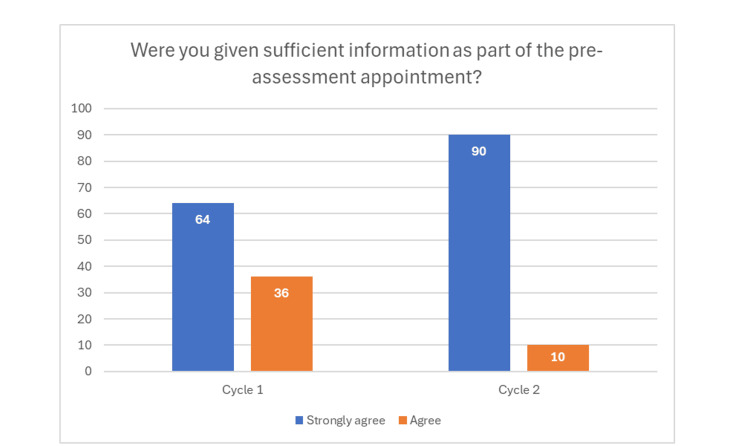
Bar chart showing improvement from cycle 1 to cycle 2, where 90% of patients strongly agreed that they were provided sufficient information about the sedation appointment from the pre-assessment telephone consultation.

In both cycles, 100% of patients felt they had the opportunity to ask questions. When asked if they felt they had been given sufficient information as part of the pre-assessment appointment, in cycle 1, 64 strongly agreed and 36 agreed, compared to 90 who strongly agreed and 10 who agreed in cycle 2, showing a 41% increase in patients feeling sufficiently informed before their sedation appointment, as highlighted in Figure 4.

**Figure 5:**
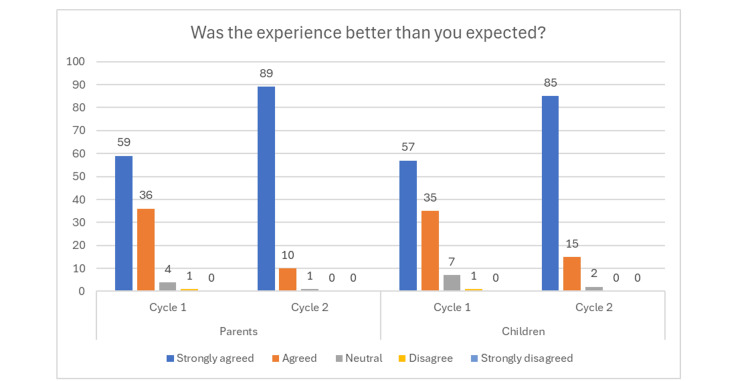
Bar chart showing improvement from cycle 1 for both parents and patients, when asked if the experience was better than expected.

In cycle 1, 59 parents strongly agreed, 36 parents agreed, four parents were neutral and one parent disagreed that their experience was better than they had expected, compared with cycle 2, where 89 parents strongly agreed, 10 parents agreed and one parent was neutral. When asked the same question to the children, in cycle 1, 57 strongly agreed, 35 agreed, seven were neutral and one child disagreed; in cycle 2, 85 strongly agreed, 15 agreed and two were neutral that their experience had been better than they had anticipated (Figure 5).

**Figure 6:**
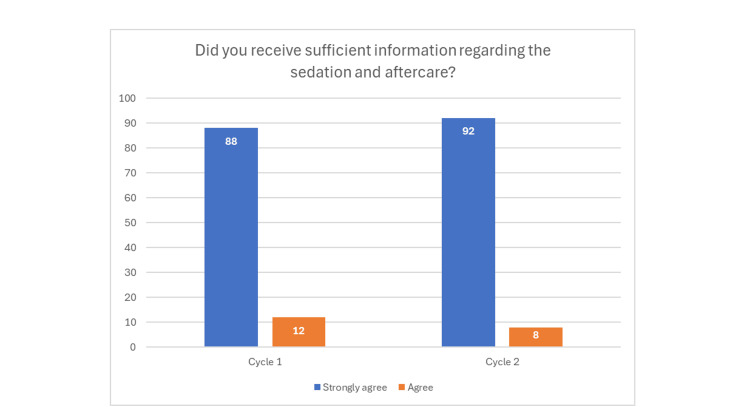
Bar chart showing improvement from cycle 1 to cycle 2, regarding information provided on the day of the treatment appointment.

In cycle 1, 88 strongly agreed and 12 agreed that they were given sufficient information regarding sedation and aftercare on the day of the treatment, compared to cycle 2, in which 92 strongly agreed and eight agreed (Figure 6).

In cycle 1, 72 strongly agreed and 28 agreed that they were seen in a child-friendly environment, compared to 100 who strongly agreed in cycle 2. When asked if they felt the sedation was helpful in their child’s treatment, in cycle 1, 92 found it very helpful, five found it somewhat helpful and three did not find it helpful. In cycle 2, 94 found it very helpful, five found it somewhat helpful and one did not find it helpful. In cycle 1, 93 said they would consider inhalation sedation for any future treatment for their child again, five were not sure and two said they would not. In cycle 2, 97 said they would consider sedation again, and three were not sure.

Thematic analysis was undertaken to review themes in the free-text part of the questionnaire. In the first cycle, seven parents commented on the smell of the sedation mask being unpleasant. In the second cycle, there were 27 positive comments written by parents about the supportive aids introduced, focusing on the scented pens and tooth pots. Common themes included how much the children enjoyed receiving the tooth pots, and how being able to choose their own scented pens added to the overall positive experience. Parents felt the scented pens were 'very helpful' in their child's co-operation, and that the tooth pots were a great adjunct to creating a child-friendly environment.

In addition to this, following cycle 2, clinicians found that many parents and children were verbally commenting on how beneficial they found the teddy bear, tooth pots and scented pens. When asked, 100% of the operative team found the supportive aids introduced to be beneficial when providing treatment under inhalation sedation. The team found that allowing children to choose their own scent from the beginning added an element of excitement for the child and improved their co-operation, allowing the placement of the sedation mask. They also felt the tooth pots were beneficial in being used as positive reinforcement throughout the treatment.

## Discussion

The results of the study highlighted that showing patients a pre-visit video and supportive aids, in addition to inhalation sedation - in particular, scented pens, tooth pots for extractions and the use of a teddy bear with a matching sedation mask to explain the treatment to the child - significantly improved parent and child satisfaction. Incorporating the items into routine practice creates a more child-friendly environment, leading to an overall improvement in patient and parent experiences. These additions were also welcomed by clinicians, who found them beneficial in their practice.

Pre-visit video

Following the COVID-19 pandemic, our pre-assessments are routinely carried out over the phone, which means that the first time many patients come to our practice is on the day of their treatment. This, alongside the fact that many children have never had dental treatment, can exacerbate the fear of unknown anticipated danger [11]. Video modelling can also be effective at reducing dental anxiety and improving the acceptance of nasal mask administration [12,13]. A pre-visit video had been previously made by our practice, showing what to expect on the day of inhalation sedation treatment; however, it was not routinely used as a pre-assessment and consenting tool. There was an increase in the number of patients who watched the pre-visit video in the second cycle following its implementation into the pre-assessment template. This subsequently led to a significant increase in parents feeling they had been given sufficient information as part of the pre-assessment. Other benefits of modelling videos are that they can be watched multiple times in the comfort of patients’ own homes prior to coming in, as well as saving clinical time and improving dentist-patient communication [12,14].

Scented pens

The unpleasant smell of the sedation mask can be a frustrating factor for paediatric inhalation sedation treatment [15], which was also found following results from cycle 1. Studies [16,17] have shown that scented anaesthesia masks facilitate mask acceptance and reduce pre-op anxiety. Before placement of the mask, giving children a choice of different scented flavours - strawberry, watermelon, banana and cola - also gives them a sense of control in their treatment, which improves compliance and co-operation, as well as reducing anxiety [18]. Following the introduction of the scented pens in the second cycle, many parents felt it had been a useful adjunct in their child's treatment and had led to an overall significant improvement in satisfaction.

Teddy bear and tooth pots

Following the first cycle, we aimed to create a more child-friendly environment with the introduction of additional aids and toys [19]. A teddy bear with a nasal mask matching the one to be worn by the patient was placed on the chair upon the arrival of the children. It was used as part of an explanation to the patient using tell-show-do and modelling [18], which made the patient more willing to accept the mask. Furthermore, tooth pots were used as positive reinforcement at the beginning of the appointment, encouraging children to choose a coloured pot and hold onto it until the end, when we would put their tooth in it to take home for the tooth fairy. In cycle 2, these interventions led to all parents strongly agreeing that their children were seen in a child-friendly environment, with a significant increase in parents and patients strongly agreeing that their experience was better than expected.

Limitations

One limitation of this study is that it did not assess previous dental experiences of participants, which can play a role in dental anxiety and patient satisfaction. A pre-assessment anxiety questionnaire for children may have been useful to help illustrate their baseline level of anxiety and to help identify adjuncts that would be useful for them. Further studies can be done to take this into consideration.

Although a large sample size was used, obtaining responses from a more diverse range of paediatric patients may have offered more useful information on how their responses differ. In the UK, paediatric patients up to the age of 16 who have complex oral health needs are referred to secondary care centres or CDS. These may be patients with severe gag reflexes, dental anxiety or poor co-operation. Once referred, they are assessed by clinicians with the same skill set as specialists or consultants, where advanced techniques such as conscious sedation using midazolam can be employed if required [8]. Carrying out a multi-centre study in primary care, secondary care and CDS units will help to determine if these interventions and adjuncts are useful for all paediatric patients receiving inhalation sedation, and how the response to these adjuncts differs depending on the patient cohort.

## Conclusions

In conclusion, a pre-visit video and additional supportive aids, in particular inhalation sedation scented pens, tooth pots for extractions and the use of a teddy bear with a matching sedation mask for the child, were found to significantly improve parent and child satisfaction. These are beneficial adjuncts and can be used alongside inhalation sedation in the management of paediatric patients and the creation of a more child-friendly environment.

## Appendices

Appendix 1 (inhalation sedation questionnaire)

Please state the age of the patient treated today

 ____________________ 

What treatment did you/your child have today? (please tick) 

Fillings 

Tooth extraction 

Both

I was given sufficient information regarding the treatment in the pre-assessment 

Strongly agree 

Agree

Neutral

Disagree

Strongly disagree

I watched the YouTube video provided in the email 

Yes 

No 

We were seen on time 

Yes 

No 

I/my child was given the chance to ask questions 

Yes 

No 

I was given sufficient information regarding sedation and aftercare

Strongly agree 

Agree 

Neutral 

Disagree 

Strongly disagree 

We were seen in a child-friendly environment

Strongly agree 

Agree 

Neutral 

Disagree 

Strongly disagree 

We were treated with respect and dignity

Strongly agree 

Agree 

Neutral 

Disagree 

Strongly disagree 

How helpful was the inhalation sedation in you/your childs’ treatment

Very helpful 

Somewhat helpful 

Not helpful 

Would you consider inhalation sedation for any further treatment you/your child requires? 

Yes 

No

Not sure 

The overall experience was better than I expected

Strongly agree

Agree

Neutral

Disagree

Strongly disagree

The overall experience was better than I had expected

Strongly agree

Agree 

Neutral 

Disagree 

Strongly disagree

The overall experience was better than my child had expected 

Strongly agree 

Agree 

Neutral 

Disagree 

Strongly disagree

Please provide any feedback

______________________________________________________________________________________________________________________________________________________________________________________________________________________________________________________________________________________________________________________________________________________________________________________________________ 
